# Cardiovascular disease prevention at the workplace: assessing the prognostic value of lifestyle risk factors and job-related conditions

**DOI:** 10.1007/s00038-018-1118-2

**Published:** 2018-05-25

**Authors:** Giovanni Veronesi, Rossana Borchini, Paul Landsbergis, Licia Iacoviello, Francesco Gianfagna, Patrick Tayoun, Guido Grassi, Giancarlo Cesana, Marco Mario Ferrario, The Cohorts Collaborative Study in Northern Italy (CCSNI) Research Group, Marco M. Ferrario, G. Cesana, R. Sega, M. T. Gussoni, F. Duzioni, L. Bolognesi, G. Veronesi, C. Fornari, L. Bertù, G. Grassi, R. Sega, G. Mancia, R. Sega, M. Bombelli, R. Facchetti, Marco M. Ferrario, A. Vivaldi, G. Veronesi, C. Fornari, L. Bertù, P. Brambilla, S. Signorini, A. Borsani, M. T. Gussoni, F. Gianfagna, S. Landone, D. Parassoni, M. Ruspa, S. Mombelli, P. de Moliner

**Affiliations:** 1 Department of Medicine and Surgery, Research Centre in Epidemiology and Preventive Medicine, University of Insubria, Varese, Italy; 2 Occupational Medicine Unit, Varese Hospital and University of Insubria, Varese, Italy; 3 Department of Environmental and Occupational Health Sciences, SUNY Downstate Medical Center, New York, USA; 4 IRCCS Neuromed, Pozzilli, Italy; 5 School of Medicine, University of Insubria, Varese, Italy; 6 Clinica Medica, University of Milano-Bicocca, Monza, Italy; 7 IRCCS Multimedica, Sesto San Giovanni, Italy; 8 Research Centre on Public Health, Department of Medicine and Surgery, University of Milano Bicocca, Monza, Italy

**Keywords:** Cardiovascular prevention, Workplace, Global workers’ health, Risk estimation, Lifestyle, Job strain, Discrimination, Clinical utility

## Abstract

**Objectives:**

The prognostic utility of lifestyle risk factors and job-related conditions (LS&JRC) for cardiovascular disease (CVD) risk stratification remains to be clarified.

**Methods:**

We investigated discrimination and clinical utility of LS&JRC among 2532 workers, 35–64 years old, CVD-free at the time of recruitment (1989–1996) in four prospective cohorts in Northern Italy, and followed up (median 14 years) until first major coronary event or ischemic stroke, fatal or non-fatal. From a Cox model including cigarette smoking, alcohol intake, occupational and sport physical activity and job strain, we estimated 10-year discrimination as the area under the ROC curve (AUC), and clinical utility as the Net Benefit.

**Results:**

*N* = 162 events occurred during follow-up (10-year risk: 4.3%). The LS&JRC model showed the same discrimination (AUC = 0.753, 95% CI 0.700–0.780) as blood lipids, blood pressure, smoking and diabetes (AUC = 0.753), consistently across occupational classes. Among workers at low CVD risk (*n* = 1832, 91 CVD events), 687 were at increased LS&JRC risk; of these, 1 every 15 was a case, resulting in a positive Net Benefit (1.27; 95% CI 0.68–2.16).

**Conclusions:**

LS&JRC are as accurate as clinical risk factors in identifying future cardiovascular events among working males. Our results support initiatives to improve total health at work as strategies to prevent cardiovascular disease.

## Introduction

In Europe, cardiovascular disease (CVD) accounts for 45% of total deaths, and for 29% of deaths before the age of 65 according to the latest epidemiological data (Townsend et al. 2016). The World Health Organization estimates that about 80% of premature ischemic heart disease and stroke is preventable (WHO 2016), especially so when primordial and primary prevention begin early in life (Weintraub et al. 2011). The maintenance of healthy lifestyles throughout young adulthood increases the prevalence of the “ideal cardiovascular health” profile in middle age (Liu et al. 2012) and substantially reduces the lifetime risk of disease (Lloyd-Jones et al. 2007). The working population comprises about 70–80% of young- and middle-aged adults in Europe (OECD 2016; Eurostat 2017). Interventions at the workplace promoting healthy diet, physical activity (PA) and tobacco control, and addressing alcohol abuse, have been recommended to reduce the risk at a population level (Piepoli et al. 2016). Besides lifestyle behaviors, a number of job-related individual exposures including perceived work stress, low occupational physical activity (OPA), long working hours and shift work have been consistently associated with cardiovascular mortality and morbidity by systematic reviews and meta-analyses (Fishta and Backé 2015; Li and Siegrist 2012; Kivimäki et al. 2015; Vyas et al. 2012). Recent literature supports the so-called OPA paradox, with increased CVD risk of high-intensity physical activity at work (Holtermann et al. 2018) and, more relevant for preventive purposes, the combined effect of OPA and sport PA during leisure time (Ferrario et al. 2018). Despite this compelling evidence, the prognostic utility of individual lifestyle and job-related conditions for CVD risk stratification in the working population has not been jointly evaluated to date in prospective studies.

In this paper, we investigated the discrimination ability and the clinical utility of lifestyle (cigarette smoking, alcohol consumption and sport PA) and job-related risk factors (job strain and OPA) in a Northern Italian working male population representing a variety of job titles. Discrimination was estimated in the overall sample and contrasted to a conventional risk stratification model, while clinical utility was investigated among workers classified at low CVD risk by current guidelines.

## Methods

### Study population

The MONICA-Brianza (two consecutive surveys, with recruitment in 1989–1990 and 1993–1994) and the PAMELA study (one survey recruited in 1990–1991) are population-based prospective cohorts of 25- to 64-year-old residents in the Brianza area, located north of Milan (Ferrario et al. 2011; Cesana et al. 1991). The Study of the Employees in the Municipality of Milan (SEMM; Ferrario et al. 2008) enrolled a random sample of civil servants between 1993 and 1996. The MONICA, PAMELA and SEMM studies have been conducted by a unique team of researchers, with harmonized procedures for risk factor assessment at recruitment and for follow-up. The studies were approved by the local ethical committees. No written informed consent signed by participants was required at the time of recruitment. Participation rates were 70.1 and 67.2% for the MONICA surveys, respectively; 64% for the PAMELA study; and 75% for the SEMM study. The present study is a pooled analysis of individuals who were employed at the time of recruitment.

### Lifestyle and job-related risk factors

Height and weight were measured on subjects without shoes and wearing light clothing during the clinical examination. Individuals were classified as normal weight (body mass index, BMI, < 25 kg/m^2^); overweight (BMI between 25 and 29.9) and obese (BMI ≥ 30 kg/m^2^). Usual physical activity (PA) at work and during leisure time was investigated using the Baecke Questionnaire (Baecke et al. 1982), a reliable tool to assess habitual PA in observational studies (Jacobs et al. 1993). Three OPA categories (“low”, “intermediate” and “high”) were derived from the original score categorized into sample tertiles. Items investigating sport activities during leisure time allowed the quantification of the absolute intensity based on metabolic equivalent of task (Strath et al. 2013) as well as of the duration in “minutes per week” of the activity. The study variable is based upon the American Heart Association categories of poor (0 min/week of activity); intermediate (1–149 min/week moderate or 1–74 min/week vigorous or 1–149 min/week moderate + vigorous activity); and recommended (≥ 150 min/week moderate or ≥ 75 min/week vigorous or ≥ 150 moderate plus vigorous activity) sport physical activity (Lloyd-Jones et al. 2010). Daily cigarette smoking and alcohol intake were investigated using self-reported questionnaires. The study variable for smoking habit is current versus non-current smoker. Daily alcohol intake (in grams) was converted to average drinks per day, considering 12.5 grams of alcohol as a standard drink (Corrao et al. 2004). We further categorized alcohol intake as abstainers (less than 0.5 drinks per day), 1–3, 4–6, and 6 or more drinks per day. The Job Content Questionnaire (JCQ; Karasek 1979) was administered to all workers. The “psychological job demand” and “job decision latitude” scores were derived from the items satisfying a preliminary construct validity assessment, as previously detailed (Ferrario et al. 2017). We derived the four JCQ categories (low strain, active, passive and high strain) based on the conventional quadrant approach using sample medians as cutoff values for psychological job demand and job decision latitude.

### Clinical risk factors

Blood pressure was measured on sitting subjects at rest for at least 10 min, using a standard mercury sphygmomanometer equipped with larger cuff bladders, if needed. The study variable for systolic blood pressure is the average of two measurements taken 5 minutes apart. Venous blood specimens were taken from the antecubital vein in fasting subjects (12 h or more). Serum total cholesterol and HDL-cholesterol were measured by an enzymatic method. Blood glucose was determined on the same samples by an enzymatic method. Diabetes was defined as either blood glucose > 126 mg/dl, or positive anamnestic information self-reported by the subject.

### Follow-up procedures

The primary study endpoint is the occurrence of a first major acute coronary event (myocardial infarction, acute coronary syndrome) or coronary revascularization, or first ischemic stroke or carotid endarterectomy. Fatal events were defined from death certificates with underlying causes of deaths (ICD-IX codes) 410–414 (coronary event) or 430–438 (ischemic strokes). Non-fatal events were identified according to the following hospital discharge ICD-IX codes: 410–411 (coronary events), 36.0–9 (coronary revascularization), 430–432, 434, 436 and 38.01–39.22 (acute strokes) and 39.50–39.52 (carotid endarterectomies). Acute events were further adjudicated according to the MONICA diagnostic criteria (MONICA Manual 1999). The ischemic stroke subtype was attributed on the available clinical information. The follow-up period ended on December 31, 2008, for all the study cohorts, and it was truncated at 15 years to take into account the different recruitment periods between the population-based (1990–1993) and the factory-based (1993–1996) cohorts. The follow-up completion was 99 and 96.8% for fatal and non-fatal events, respectively.

### Statistical analysis

The study cohorts totaled *n* = 3088 men and *n* = 3754 women, 35–64 years old, employed and with no previous cardiovascular disease at recruitment visit. Due to the low number of CVD events during follow-up (*n* = 58), women were not further analyzed. We excluded 553 men due to missing data on clinical, lifestyle and job-related risk factors, leaving a final sample size of 2532. Excluded workers had a similar cumulative 10-year risk of CVD as complete cases (4.1 vs. 4.3%; log-rank test *p* value = 0.98). Lifestyle and job-related conditions were summarized by descriptive statistics, in the overall sample and by current smoking status, since smoking is included in the conventional CVD risk scores (Piepoli et al. 2016, Table 2). Differences between smokers and non-smokers were tested using either a t test or a Chi-square test, for continuous and discrete variables, respectively. We observed a significant interaction between occupational and sport PA (Wald Chi-square test *p* value for interaction: 0.01), confirming previous findings (Ferrario et al. 2018), and we constructed a six-level combined PA as the main study variable. We tested the proportionality of the hazards hypothesis for every variable by running separate Cox models with a variable*time interaction. Proportionality was confirmed for all the variables (all *p* values > 0.05). Lifestyle and job-related risk factors satisfying the Akaike information criterion (Steyerberg 2009) in a multivariable Cox regression model adjusted for age and cohort type constituted the “lifestyle and job-related condition” (LS&JRC) model. The discrimination ability at 10 years for the LS&JRC model was estimated as the area under the ROC curve (Chambless et al. 2011), and contrasted to a conventional risk score including total cholesterol (4 categories: less than 200 mg/dl, 200–239, 240–279, ≥ 280 mg/dl), HDL-cholesterol (4 categories: less than 45, 45–49, 50–59, ≥ 60 mg/dl), smoking, systolic blood pressure (continuous) and diabetes. Such a conventional risk score was recently developed (Veronesi et al. 2013) and validated (Veronesi et al. 2015) on the Italian population. The overlap of 95% confidence intervals for the AUCs, estimated using bias-corrected bootstrapping, represented no statistically significant difference in discrimination between the two models. We estimated the change in AUC from a reference model including only age and smoking, to disentangle the contribution of the remaining risk factors.

Finally, we investigated the potential for clinical utility of the LS&JRC model among workers at low CVD risk according to guidelines, i.e., with predicted 10-year risk of CVD mortality from the European SCORE equation less than 1% (Piepoli et al. 2016). We used the published SCORE coefficients for low-risk countries (Conroy et al. 2003) and recalibrated the overall survival to the observed mortality in our study sample, as recommended. The recalibrated SCORE had a 10-year discrimination of 0.681 in the overall sample. We estimated sensitivity, specificity, the Number Needed to Screen in order to identify a future case, and the Net Benefit (Vickers et al. 2016) for the LS&JRC model, when using the observed 10-year risk of CVD as a threshold value to define true and false positives. These parameters were also estimated among workers with normal levels of clinical risk factors, i.e., total cholesterol < 240 mg/dl; systolic blood pressure < 140 mmHg and no treatment; no diabetes. The analyses were performed using the Statistical Analysis System (9.4 release; SAS Institute, Cary, North Carolina, USA).

## Results

We included 2532 workers, mean age 45.5 ± 7.2, 13% executives, 38% non-manual workers, 40% manual workers and 9% self-employed. The distribution of lifestyle risk factors and job-related conditions in the study sample is summarized in Table 1. Optimal levels of lifestyle risk factors were observed in about 40% of the sample: BMI less than 25 kg/m^2^ (36%), alcohol intake 1–3 drinks/day (39%), any sport PA (intermediate, 18%; recommended, 13%). Sedentary workers were 41%, while 30% were in the high OPA category. Finally, high job strain was present in 25% of workers, as expected under the JCQ median-cutpoint formula. Body mass index and job strain were not associated with smoking status (Table 1). Conversely, current smokers were more likely to be heavy drinkers (12 vs. 7%) and physically inactive (76 vs. 64%), and less likely to be sedentary workers (37 vs. 43%) than non-current smokers (all *p* < 0.05).

**Table 1:** Distribution of lifestyle risk factors and job-related conditions at baseline, in the pooled sample and by smoking status. Brianza (Northern Italy), 1989–1996

	Pooled sample	Smoking status		*p* value
		Current smokers	Non-current smokers	
*N*	2532	972	1560	–
Age, years	45.5 (7.2)	45.2 (7)	45.7 (7.3)	0.12
Body mass index, %				
≤ 25 kg/m^2^	36.0	38.4	34.5	0.10
≤ 30 kg/m^2^	48.9	47.7	49.6	
> 30 kg/m^2^	15.1	13.9	15.9	
Daily alcohol intake, %				
None	33.0	29.0	35.4	0.0001
1–3 drinks/day	39.1	38.6	39.5	
4–6 drinks/day	19.0	20.8	17.8	
6+ drinks/day	9.0	11.7	7.2	
Occupational PA^a^, %				
Low	40.8	37.1	43.1	0.01
Intermediate	29.8	31.6	28.7	
High	29.4	31.3	28.2	
Sport PA^b^, %				
Poor	68.8	76.2	64.2	< .0001
Intermediate	18.0	14.8	20.0	
Recommended	13.2	9.0	15.8	
Job strain				
Low	24.7	22.5	26.1	0.06
Active	14.3	14.2	14.4	
Passive	36.4	36.1	36.6	
High strain	24.5	27.2	22.9	

In the table: mean (SD) for continuous variables, % for categorical variables

*P* value testing the null hypothesis of no association between smoking status and other lifestyle risk factors and job-related conditions. *t* test for continuous variables, Chi-square test for categorical variables

*PA* physical activity

^a^Categories defined according to sample tertiles

^b^Categories defined by the American Heart Association [20]. Poor: 0 min/week of activity; intermediate: 1–149 min/week moderate or 1–74 min/week vigorous or 1–149 min/week moderate + vigorous activity; recommended: ≥ 150 min/week moderate or ≥ 75 min/week vigorous or ≥ 150 moderate + vigorous activity

During follow-up (median 14 years), 162 first coronary heart disease or ischemic stroke events occurred, corresponding to a cumulative 10-year risk of CVD of 4.3%. There was no difference in cumulative 10-year risk according to study cohort type (population- vs. factory-based; log-rank test *p* value: 0.17). In univariate Cox regression models adjusted for age and study type, body mass index was not associated with the endpoint (Table 2). The remaining variables met the Akaike information criterion and entered the final LS&JRC model: smoking (hazard ratio, HR = 2.47, 95% CI 1.80–3.40); alcohol intake (abstainers: 1.53, 1.04–2.26; 6 + drinks/day: 1.67, 1.00–2.77); high job strain (1.75, 1.11–2.78); combined occupational and sport PA, as sport PA had a protective effect in sedentary workers (0.41, 0.22–0.76), but a harmful effect among workers at high OPA levels (1.56, 0.81–2.98; Wald Chi-square test for interaction *p* value = 0.01).

**Table 2:** Number of events, event rates, univariate and multivariate association between lifestyle risk factors and job-related conditions (LS&JRC) with the incidence of coronary heart disease or ischemic stroke events. Brianza (Northern Italy), 1989–2008

	*N*	# events	Rate	Model 1		Model 2	
				Hazard ratio (95% CI)	*p* value^a^	Hazard ratio (95% CI)	*p* value^a^
Current smoking							
No	1560	67	3.1	REF	< .0001	REF	< .0001
Yes	972	95	7.7	2.54 (1.86; 3.47)		2.47 (1.80; 3.40)	
Body mass index							
≤ 25 kg/m^2^	911	54	4.9	REF	1.0	–	–
≤ 30 kg/m^2^	1238	82	4.8	1.00 (0.71; 1.42)		–	
> 30 kg/m^2^	383	26	4.8	1.02 (0.64; 1.63)		–	
Daily alcohol intake							
None	835	53	5.4	1.40 (0.95; 2.06)	0.12	1.53 (1.04; 2.26)	0.10
1–3 drinks/day	991	51	3.8	REF		REF	
4–6 drinks/day	480	33	4.8	1.23 (0.79; 1.92)		1.27 (0.81; 1.98)	
6+ drinks/day	226	25	7.2	1.77 (1.06; 2.93)		1.67 (1.00; 2.77)	
Combined occupational and sport PA							
Low OPA, poor sport PA	654	62	6.7	REF	0.01	REF	0.01
Low OPA, intermediate/recommended sport PA	379	12	2.5	0.36 (0.20; 0.68)		0.41 (0.22; 0.76)	
Intermediate OPA, poor sport PA	510	28	4.0	REF		REF	
Intermediate OPA, intermediate/recommended sport PA	245	8	3.3	0.86 (0.39; 1.90)		1.00 (0.45; 2.23)	
High OPA, poor sport PA	578	40	5.1	REF		REF	
High OPA, intermediate/recommended sport PA	166	12	6.6	1.29 (0.67; 2.46)		1.56 (0.81; 2.98)	
Job Strain							
Low strain	626	34	3.6	REF	0.05	REF	0.11
Active	363	24	5.0	1.48 (0.87; 2.51)		1.43 (0.84; 2.43)	
Passive	922	59	4.7	1.38 (0.90; 2.12)		1.28 (0.83; 1.96)	
High strain	621	45	6.2	1.91 (1.21; 3.03)		1.75 (1.11; 2.78)	

Rate: age-adjusted event rate, per 1000 person-years, estimated at the sample mean age of 45. Model 1: Univariate Cox regression models, adjusted by age and study type (population- vs. factory-based cohort). Model 2: Cox regression model including all LS&JRC except body mass index, adjusted by age and study type (population- vs. factory-based cohort)

*PA* physical activity, *CI* confidence interval

^a^Wald Chi-square test (df equal to the number of variable levels minus 1). Akaike information criterion *p* value: 0.157 for 1 df test (smoke); 0.135 for 2 df test (BMI); 0.112 for 3 df tests (alcohol, job strain), 0.075 for 5 df test (combined occupational and sport PA)

Models’ calibration and discrimination are reported in Table 3. All models were well calibrated (Gronnesby–Borgan test *p* values > 0.05). The full LS&JRC model (M4 in the table) had a discrimination of 0.753 (95% CI: 0.700–0.780). Altogether, occupational and sport PA, alcohol intake and job strain increased the AUC by almost 3% (0.028; 95% 0.011–0.040) from the referent model (age and smoking only; M1). The addition of either lifestyle risk factors (alcohol intake, sport PA; M2) or of job-related conditions (OPA and job strain; M3) increased the AUC by about 1% each from the reference. The conventional risk model (M5) had the same discrimination ability (0.753; 95% CI 0.713–0.779) as the final LS&JRC model. This finding was consistent across all the occupational classes (Table 4), as well as by cohort type (population- vs. factory-based cohorts; data not shown). Altogether, the change in AUC led by total- and HDL-cholesterol, systolic blood pressure and diabetes over the reference model was of similar magnitude (0.029) than the one due to PA, alcohol intake and job strain.

**Table 3:** Calibration and discrimination ability at 10 years for incident cardiovascular disease risk estimation models based on lifestyle risk factors and job-related conditions (LS&JRC), and for a conventional risk model including blood lipids, blood pressure, smoking and diabetes. Brianza (Northern Italy), 1989–2008

	Calibration	Discrimination^a^			
		AUC (95% CI)		Δ-AUC^b^ (95% CI)	
M1: age, smoking status	10.9	0.724	(0.684; 0.759)	REF	
M2: M1 + sport PA, alcohol consumption	6.0	0.734	(0.692; 0.764)	0.010	(0.002; 0.019)
M3: M1 + occupational PA, job strain	16.6	0.736	(0.691; 0.767)	0.012	(0.004; 0.021)
M4: M1 + combined occupational and sport PA, alcohol consumption and job strain	14.3	0.753	(0.700; 0.780)	0.028	(0.011; 0.04)
M5: M1 + total cholesterol, HDL-cholesterol, systolic blood pressure and diabetes	10.7	0.753	(0.713; 0.779)	0.029	(0.012; 0.044)

Calibration: Gronnesby–Borgan goodness-of-fit Chi-square value. A value below 17 is considered indication of model fit

All models additionally include a dummy variable to indicate study type (population- vs. factory-based cohort)

*AU*C area under the receiver operating characteristic curve, *CI* confidence interval, *PA* physical activity, *HDL* high-density lipoprotein

^a^Evaluated at 10 years. 95% CI from bootstrapping (*n* = 2000 runs)

^b^From Model 1

**Table 4:** Area under the receiver operating characteristic curve for cardiovascular disease risk estimation model based on lifestyle risk factors and job-related conditions (LS&JRC) and for a conventional risk model including blood lipids, blood pressure, smoking and diabetes, in different occupational classes. Brianza (Northern Italy), 1989–2008

	Executives (*n* = 325, CVD = 27)	Non-manual workers (*n* = 973, CVD = 53)	Manual workers (*n* = 1010, CVD = 57)	Self-employed (*n* = 224, CVD = 25)
M1: age, smoking status	0.716	0.716	0.720	0.724
M1 + combined occupational and sport PA, alcohol consumption and job strain	0.750	0.756	0.740	0.742
M1 + total cholesterol, HDL-cholesterol, systolic blood pressure and diabetes	0.739	0.746	0.753	0.751

*HDL* high-density lipoprotein, *CVD* cardiovascular disease

In the table: Area under the receiver operating characteristic curve (AUC) evaluated at 10 years, in different working categories. Models’ coefficients were estimated in the whole sample, and the AUC was estimated in each working category. All models additionally include a dummy variable to indicate study type (population- vs. factory-based cohort)

According to European guidelines, *N* = 1832 workers were classified at “low CVD risk”; these experienced 91 events during follow-up, corresponding to a cumulative 10-year risk of 3.3% (Table 5). The discrimination ability for the LS&JRC model in these workers was satisfactory (AUC = 0.745). *N* = 687 workers in this group (38%) had LS&JRC risk greater than 3.3%; at this cutoff value, the score had 75% sensitivity and 64% specificity, and 1 out of every 15.2 men experienced a CVD event in 10 years. The Net Benefit was greater than zero (1.27; 95% CI: 0.68–2.16). Similar figures were obtained when considering workers without established clinical risk factors (Table 5).

**Table 5:** Discrimination and clinical utility parameters for the risk estimation model based on lifestyle risk factors and job-related conditions (LS&JRC), among workers without clinical CVD risk factors or at low CVD risk according to guidelines. Brianza (Northern Italy), 1989–2008

	*N*	# events	Observed 10-years risk	AUC	Workers with LS&JRC risk above the observed 10-year risk^a^					
					*N*	%	Sensitivity	Specificity	NNS	Net Benefit (95% CI)
Total cholesterol < 240 mg/dl	1811	95	3.4	0.754	695	38.4	0.719	0.628	15.6	1.19 (0.57; 2.01)
Systolic BP < 140 mmH, no treatment	908	51	3.3	0.743	406	44.7	0.830	0.566	16.2	1.32 (0.47; 2.78)
No diabetes	2447	145	4.0	0.753	822	33.6	0.665	0.678	12.6	1.38 (0.64; 2.05)
Low CVD risk^a^	1832	91	3.3	0.745	687	37.5	0.746	0.638	15.2	1.27 (0.68; 2.16)

10-year predicted CVD risk from the SCORE model < 1% and no diabetes

LS&JRC model: including age, alcohol intake, combined occupational and sport PA, smoking, job strain. The model additionally includes a dummy variable to indicate study type (population- vs. factory-based cohort)

*NNS* number needed to screen in order to identify 1 future CVD case within 10 years

^a^Observed 10-year risk in the group, as estimated from Kaplan–Meier (column 4 in this table)

## Discussion

In this Northern Italian working male population characterized by a variety of job titles, lifestyle risk factors and job-related conditions had a discrimination ability of 0.753 (95% CI: 0.700–0.780). In a large cohort of US male health professionals, lifestyle risk factors, including diet, the authors estimated an AUC at 10 years of 0.77, thus comparable to ours (Chiuve et al. 2014). The LS&JRC model had the same discrimination ability as a conventional risk prediction model including blood lipids, blood pressure, smoking and diabetes. Altogether, alcohol intake, occupational and sport PA, and job strain added 3% to discrimination over the contribution of age and cigarette smoking, the same as blood lipids, blood pressure and diabetes. All considered, these findings suggest that lifestyle risk factors and work-related conditions are as accurate as clinical risk factors in identifying CVD-free workers who will develop a major CVD event, but potentially at a lower cost of screening. In addition, almost three out of four workers in our sample were classified at very low CVD risk by the ESC-SCORE model and therefore do not qualify for any pharmacological intervention. These accounted for 56% of the CVD events during follow-up. Similarly, in a recent cross-sectional survey of Dutch employees (van der Hoeven et al. 2015), only 4.3% had SCORE ≥ 5%. The risk score based on lifestyle and job-related conditions identified 40% of low-risk workers with expected risk higher than the average: One out of every 15.2 experienced a major CVD event in 10 years. The estimated Net Benefit greater than zero means that the LS&JRC model has a positive net balance between the benefit of case identification and the harm of unnecessary screening (Vickers et al. 2016). These clinical utility parameters indicate that lifestyle risk factors and job-related conditions may help identifying workers who could benefit most by interventions occurring early in the disease process, potentially preventing disease progression and reducing healthcare costs. Literature suggests that non-pharmacological interventions at the workplace are more effective in reducing the cardiometabolic risk when targeted to selected workers with un-safe health profile (Groeneveld et al. 2010; Thorndike 2011). In addition, worksite interventions may lead to a reduction in healthcare costs between 18% and 26%, with $3.3 to $6.0 estimated saving for every dollar invested (Arena et al. 2013).

In recent years, there has been an increasing interest toward the concept of worksite health promotion and wellness as a strategy to reduce the burden of cardiovascular diseases in Europe as well as in the USA (Guazzi et al. 2014; Cahalin et al. 2014). However, CVD prevention guidelines have so far been limited their recommendations to interventions promoting individual health-related behaviors: encouraging smoking cessation, preventing alcohol abuse and favoring access to physical activity and to a healthier diet. In our analysis, when considered separately, lifestyle risk factors and job-related conditions provided the same contribution to the improvement in discrimination (see Model 2 and Model 3 in Table 3). When considered together (Model 4), the change in AUC toward the reference model was more than the sum of the two separate contributions. Therefore, our findings highlight the importance for CVD prevention of a comprehensive program which also includes interventions designed to produce a healthier work organization, to reduce job strain, to encourage physical activity for sedentary workers (Buckley et al. 2015) and to reduce strenuous OPA (Straker et al. 2017). Such an integrated approach has been recommended to prevent chronic diseases at the workplace (Sorensen et al. 2011); the Total Worker Health© initiative by the U.S. National Institute for Occupational Health and Safety represents a recent exemplification (Schill and Chosewood 2013).

Among the study limitations, we restricted our analyses to men only, due to the low number of events among women. We did not have data on other specific job exposures which have been associated with cardiovascular disease, such as effort–reward imbalance, long working hours or noise. Job strain and occupational physical activity were self-reported using validated questionnaires, a well-recognized standard in large epidemiological studies including population-based cohorts. We previously reported satisfactory construct validity and internal consistency for the JCQ items (Ferrario et al. 2017) and for the Baecke questionnaire (Ferrario et al. 2018). Self-reported information on smoking and alcohol intake was potentially underreported, in particular in the factory-based sample. Furthermore, the available data did not allow a more precise characterization of the drinking pattern for all the study cohorts, but the investigated populations at the time of recruitment were mostly characterized by a habitual daily alcohol intake often during meals. The increased CVD risk of non-drinkers as well as of heavy drinkers that we report in Table 2 is similar to what has been reported in other observational studies (Corrao et al. 2004). We only have one measurement of risk factors at baseline. Job-related risk factors may have changed during follow-up upon modifications of the working conditions or retirement. Occupational PA is related to job title, which is relatively stable over the working life. The effects of a cumulative exposure to low- or high-intensity PA at work may persist after retirement (Straker et al. 2017) and determine the observed long-lasting association between CVD risk and occupational PA (Ferrario et al. 2018). In a longitudinal study setting, cumulative job strain exposure had a stronger association with CVD risk than a single assessment (Chandola et al. 2008), suggesting that we may have underestimated the true association. Finally, we did not find any meaningful difference in the hazard ratios for high job strain in 35–54 vs. 55–64 years old (interaction test *p* value: 0.9), in agreement with previous observations on long-term effects of job strain on CVD risk (Emeny et al. 2013, Ferrario et al. 2017). Finally, due to the lack of longitudinal measurement of risk factors, in our definition of “false positive,” we potentially included workers who did not experience any event because of initiation of drug therapy during follow-up. However, the clinical utility analysis focused on workers with very low estimated baseline CVD risk (< 1%), whose probability of treatment initiation during follow-up is fairly low.

Among the study strengths, our findings come from prospective cohort studies with harmonized procedures to collect baseline and follow-up data. Participation rates were > 65% in all the study cohorts. The event adjudication using MONICA criteria was consistent over time, and loss to follow-up was very low both for fatal and for non-fatal events. The study sample comprises a variety of job titles, and the consistency of discrimination across occupational classes increases our confidence in the generalizability of the findings.

To conclude, in our working male population, lifestyle and job-related conditions had the same discriminant ability as clinical risk factors in identifying future cardiovascular events but potentially at a lower cost of screening, and they may help identifying workers who could benefit most by early preventive interventions. These findings call for future studies specifically investigating the cost-effectiveness of alternative strategies for CVD screening in the working population. Our results support initiatives to promote total or global health at work (Sorensen et al. 2011; Schill and Chosewood 2013) as strategies to prevent cardiovascular disease, and in particular the implementation of trials including and comparing efforts to improve unhealthy lifestyles and to create a healthier work organization.
